# Needle-free, spirulina-produced *Plasmodium falciparum* circumsporozoite vaccination provides sterile protection against pre-erythrocytic malaria in mice

**DOI:** 10.1038/s41541-022-00534-5

**Published:** 2022-10-04

**Authors:** Tracy Saveria, Chaitra Parthiban, Annette M. Seilie, Colin Brady, Anissa Martinez, Ridhima Manocha, Esha Afreen, Hui Zhao, Ashley Krzeszowski, Jeremy Ferrara, Troy Paddock, James Roberts, Brad C. Stone, Michael Tasch, Sean C. Murphy

**Affiliations:** 1 Lumen Bioscience, Inc., Seattle, WA 98109 USA; 2 Department of Laboratory Medicine and Pathology, University of Washington, Seattle, WA 98109 USA; 3 Center for Emerging and Re-emerging Infectious Diseases, University of Washington, Seattle, WA 98109 USA; 4 Department of Microbiology, University of Washington, Seattle, WA 98109 USA

**Keywords:** Protein vaccines, Malaria

## Abstract

Antibodies against the *Plasmodium falciparum* circumsporozoite protein (PfCSP) can block hepatocyte infection by sporozoites and protect against malaria. Needle-free vaccination strategies are desirable, yet most PfCSP-targeted vaccines like RTS,S require needle-based administration. Here, we evaluated the edible algae, *Arthrospira platensis* (commonly called ‘spirulina’) as a malaria vaccine platform. Spirulina were genetically engineered to express virus-like particles (VLPs) consisting of the woodchuck hepatitis B core capsid protein (WHcAg) displaying a (NANP)_15_ PfCSP antigen on its surface. PfCSP-spirulina administered to mice intranasally followed by oral PfCSP-spirulina boosters resulted in a strong, systemic anti-PfCSP immune response that was protective against subcutaneous challenge with PfCSP-expressing *P. yoelii*. Unlike male mice, female mice did not require Montanide adjuvant to reach high antibody titers or protection. The successful use of spirulina as a vaccine delivery system warrants further development of spirulina-based vaccines as a useful tool in addressing malaria and other diseases of global health importance.

## Introduction

*Plasmodium* parasites caused 241 million cases of malaria and 627,000 deaths in 2020, with 95% of the burden in the WHO African region^1^. Recent reports^2–4^ suggest that the COVID-19 pandemic is further exacerbating the burden of the disease and halting much of the progress made over the last 20 years. Safe and highly effective vaccines are urgently needed to re-invigorate and sustain the fight against malaria. *Plasmodium* infection is initiated by blood-feeding *Anopheles* mosquitoes that release sporozoites (spz) into the skin from where they migrate to the liver and invade hepatocytes. There, they multiply and develop into merozoites, which are released from hepatocytes into the bloodstream to initiate repeated cycles of erythrocyte infection that lead to all malaria-associated pathologies. Spz-specific antibodies are the first line of defense against infection as they limit trafficking to the liver^5^. RTS,S/AS01, the first and only WHO-approved vaccine against malaria, primarily induces spz invasion-blocking antibodies against the NANP repeat region of *P. falciparum* circumsporozoite protein (PfCSP). In a Phase 3 study, it achieved moderate efficacy with a standard vaccination course and improved outcomes with an added booster^6^. Recently, the PfCSP-based R21/MM vaccine demonstrated superior efficacy^7^, but like RTS,S, it requires refrigeration and needle-based administration, which pose logistical challenges for vaccine distribution in resource-limited areas. While imperfect, these vaccines represent important first steps toward developing effective malaria vaccines and define a role for spz invasion blocking antibodies in successful disease prevention efforts.

Despite advantages in terms of compliance, cost, delivery, and lack of medical waste, needle-free vaccines remain largely unrealized. Intranasal delivery is a recognized potent vaccination route against respiratory pathogens like influenza^8^ and SARS-CoV-2^9^, while successful oral vaccines, such as those against polio or *Salmonella typhi*, generally target pathogens that are associated with the gastrointestinal tract. These often rely on whole attenuated or inactivated organisms because subunit vaccines are less immunogenic and hence need additional components or adjuvants to induce the desired response. Subunit intranasal vaccines require optimization in terms of particle size and other physiochemical properties^10^. Experimental subunit oral vaccines have been attempted using expression systems in numerous plants, eukaryotic algae, yeasts, and bacteria. These platforms have suffered from technical and logistical challenges, including poor expression, inherent toxicity, unpalatability, poor IgG induction, and/or expensive extraction/purification procedures.

**Fig. 1:**
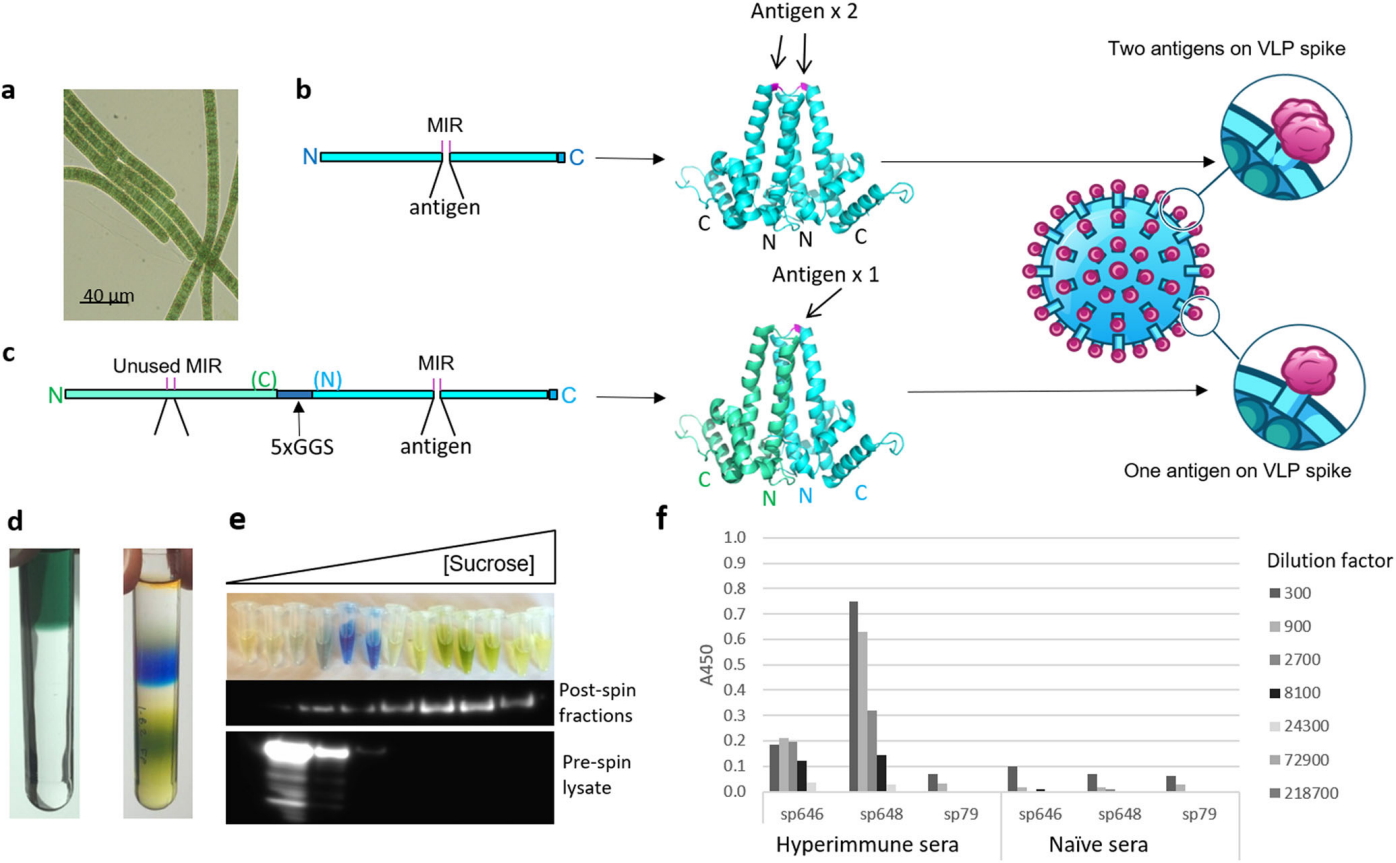
Engineering, expression, and antibody recognition of the PfCSP-spirulina VLP vaccine. **a** Light micrograph of spirulina filaments. Magnification ×400. **b**, **c** Spirulina were engineered to carry Woodchuck hepadnavirus core proteins (WHcAg) by cloning a single copy (**b**) or two copies linked in tandem by five Gly-Gly-Ser amino acid repeats (5xGGS) (**c**) of the WHcAg sequence into a plasmid with arms for homologous recombination into the spirulina chromosome. **b** Insertion of the PfCSP antigen at serine 78 in the Major Insertion Region (MIR) of the single copy construct forms dimers containing two copies of each antigen within the spike. **c** In cases where the WHcAg sequence was linked in tandem, only the second MIR site was used for placement of the PfCSP antigen, forcing the formation of dimers displaying only one antigen within the spike. **d** Spirulina strains were tested by sucrose density ultracentrifugation and fractionation to demonstrate their ability to form particles; results for sp82 shown. Pre- (left) versus post-centrifugation (right) images show the sedimentation fractions of different pigments present in spirulina, such as phycocyanin (blue). **e** 1-mL fractions were collected by bottom puncture of the tube with every other fraction resolved by SDS-PAGE and Western blotted using anti-Myc antibody (middle panel). A separate sucrose gradient was carried out on lysate following pre-treatment with SDS to show particle disruption (bottom panel). Molecular weight markers are shown in kDa. **f** PfCSP-VLPs were also screened by ELISA for their ability to be recognized by serum from BALB/cJ mice previously immunized intravenously three times with 2 × 10^4^ PfCSP-expressing *P. yoelii* spz. Bars show ELISA absorbance at 450 nm (A450) for the displayed three-fold dilutions of hyperimmune sera ranging from 1:300 to 1:218,700. Sera from naïve mice served as a control. sp646 and sp648 were PfCSP-carrying particles and sp79 was the control no-antigen particle. Images of VLP dimers were designed using PyMol software with PDB 6ECS^73^ as template.

*Arthrospira platensis* (Fig. 1a) commonly known as spirulina, offers a novel mucosal vaccine platform capable of overcoming many of the above hurdles. Spirulina is an edible, gram-negative, photosynthetic bacterium consumed worldwide for its high protein content and other benefits^11^. It has been farmed at a commercial scale as a health food since the 1970s and carries a Generally Recognized as Safe (GRAS) status from the U.S. FDA^12^. Numerous clinical trials have established its safety for consumption by adults, children, and infants^13–15^. For example, the FDA’s Center for Food Safety and Nutrition conducted a full review of the toxicology data on the organism and its traditional uncontrolled outdoor production processes and determined a no-observed-adverse-effect level for spirulina powder of 300,000 ppm, equivalent to 15 grams per kg body weight per day^12^. Genetic transformation of spirulina allows high expression of therapeutics at low cost as the biomass itself is safe for consumption and can be administered orally with or without purification^16^. Bioencapsulation within spirulina biomass protects the therapeutic cargo from the low pH and high pepsin gastric environment. Notably, protein biologics produced in spirulina are stable within the dried biomass for at least 12 months without refrigeration, facilitating stockpiling and eliminating cold-chain distribution requirements^16^. We recently reported results from a first-in-human clinical trial that tested a spirulina-based antibody therapeutic against *Campylobacter jejuni* and showed that it was safe and well tolerated at all doses tested (maximum dosage: 9 g per day for 28 days)^16^. For therapeutic purposes, spirulina can be administered as unpurified biomass powder or in an extracted formulation, the latter of which serves to concentrate soluble cytoplasmic antigens by removing insoluble material.

Here, we describe the development of a spirulina-based malaria vaccine composed of a virus-like particle (VLP) displaying the NANP repeat region of PfCSP. Prior to our studies, it was known that intraperitoneally injected woodchuck hepatitis B-antigen (WHcAg) VLPs displaying PfCSP repeat epitopes induced PfCSP-specific antibodies that sterilely protected 80–100% of mice against challenge by mosquitoes infected with hybrid PfCSP-expressing *P. berghei* spz and that *P. vivax* CSP (PvCSP) VLPs could similarly elicit sterile immunity to hybrid PvCSP-expressing *P. berghei* spz^17^. Our work here demonstrated that needle-free administration can attain similar successful outcomes and that a combination of intranasal plus oral delivery is superior to oral delivery alone for the development of NANP-specific antibodies and for protection in a PfCSP-expressing *P. yoelii* spz challenge model. To our knowledge, VLP-containing intranasal administration has not been applied to malaria vaccination.

## Results

### Manufacturing and stability of spirulina expressing VLPs bearing *Plasmodium* CSP antigens

The woodchuck hepatitis B core capsid VLP is a well described vaccine vehicle^18,19^, chosen for its tolerance for heterologous protein sequences in its outward oriented spikes following particle formation. It has been proposed to present B cells with a regular, rigid array of antigen, which results in efficient cross-linking of surface B cell receptors (BCRs) and consequent activation^20^. The PfCSP NANP_x_ antigen behaves similarly^21,22^, crosslinking clonal BCRs on a single cell. The combined effect of these regularly spaced antigens is thought to result in potent B cell activation. For this study, we cloned the WHcAg sequence into a plasmid with left and right homology arms for recombination into the spirulina chromosome and used Serine-78^23^ within the major insertion region (MIR) of WHcAg as the site for incorporation of our selected antigens (Fig. 1b). We also engineered a construct where we placed two copies of the WHcAg sequence in tandem, connected by a flexible Gly-Gly-Ser linker, and used only the MIR of the second copy for antigen insertion (Fig. 1c). This strategy allows the formation of dimers that display only one antigen at a time, alleviating the potential steric hindrance that may occur when large antigens are in close proximity^24–26^. Constructs were transformed into spirulina to generate *Plasmodium* targeting vaccine strains for inclusion in this study.

We selected two strains to be set aside for testing the long-term stability of spirulina-produced vaccines: one expressing the *P. yoelii* CSP B cell repeat epitope cloned into the MIR of the single polypeptide WHcAg sequence followed by a C-terminal T cell epitope (sp82), and one expressing the *P. falciparum* NANP repeat region within the second MIR of the tandem WHcAg sequence (sp648). Aliquots of dried biomass were stored at various temperatures, ranging from −80 to 42 °C, for 10 months (sp648) or one year (sp82), then analyzed by either an automated capillary immunoblotting system (sp648) or run on SDS-PAGE followed by traditional western blot (sp82). For sp82, anti-Myc antibody detected the reduced monomers around 35 kDa (expected size, 27 kDa) and analysis using ImageJ software showed no significant decrease in the abundance of the monomer relative to time zero (Supplementary Fig. 1). Faint bands at higher molecular weights were also detected in some samples taken from temperatures above 4 °C. To analyze sp648, we used an automated nano-immunoassay system to measure total soluble protein as a percent of the sample’s respective dry weight and additionally compared how two different methods of drying spirulina biomass, lyophilization and spray drying (Supplementary Fig. 1), might affect protein stability. Here, we used both an anti-His antibody to detect the C-terminal 6× His tag, as well as an anti-NANP antibody to detect the antigen within the MIR. Analysis of samples stored following lyophilization (1B), showed that at the highest temperatures (42 and 37 °C), a lower amount of soluble target protein was present compared to samples stored at lower temperatures (amount based on the 53kD band). This loss was greatly minimized in the samples stored at higher temperatures following our spray drying method (Supplementary Fig. 1). In concurrent studies carried out in spirulina strains expressing heavy chain antibodies, we demonstrated that binding affinity for target proteins is maintained despite long-term exposure to high-temperature conditions^16^. Taken together, these data suggest that proteins in dry spirulina biomass remain stable and bioactive over long periods at temperatures up to 42 °C.

The sp82 spirulina strain was also tested by sucrose density ultracentrifugation and fractionation to determine the ability to form particles. Here, we lysed whole cell biomass by sonication and then analyzed the soluble fraction by sucrose velocity sedimentation (Fig. 1d, left panel). Fractions (Fig. 1d, right panel) were displayed by SDS-PAGE (Fig. 1e, top panel), followed by western blotting to visualize the Myc-tagged protein monomers (Fig. 1e, middle panel). In parallel, we analyzed an SDS-treated sample of the spirulina lysate in order to disrupt particles before sedimentation (Fig. 1e, bottom panel). In the non-SDS treated lysate samples, antibody detected the strongest bands in the higher percentages of sucrose, including the 60% fractions where we observed membrane-associated fragments of chlorophyll, easily identified by their green color. Chlorophyll pigments bind proteins to form two large reaction centers called photosystems one and two (PSI and PSII, respectively) which associate with thylakoid membranes to carry out photosynthesis. Based on the sizes of these complexes, we can infer that particles at this position within the sucrose gradient are at least between 0.54 MDa^27^ and 1.06 MDa^28^, the sizes of PSII dimers and PSI trimers, respectively. The predicted size of the full sp82 particle is 6.48 MDa, which should therefore be detected at the upper range of the gradient as it was here. When fractions were run after pre-treatment with SDS, we detected the monomers in the lowest percentage sucrose fractions (bottom panel) showing that particle formation was abolished by SDS pre-treatment. These observations are consistent with what was previously reported for particle formation^29,30^.

Next, with the goal of moving our PfCSP candidates into animal studies, we verified that they could be recognized by relevant immune sera. Here, we used Enzyme-Linked Immunosorbent Assay (ELISA) to screen PfCSP-VLP strains for recognition by serum from BALB/cJ mice previously immunized intravenously three times with 2 × 10^4^ PfCSP-expressing *P. yoelii* spz (Fig. 1f). For this assay, we coated 96 well plates with the soluble protein fractions from two different PfCSP-VLP strains, one where the NANP repeat region was cloned into the MIR of the single polypeptide WHcAg sequence (sp646) and one where it was cloned into the MIR of the second WHcAg sequence in the tandem construct (sp648). We then incubated with serial dilutions of sera from hyperimmunized mice to assess affinity. Figure 1f shows that sera from these mice displayed stronger recognition of total soluble protein extracted from sp648 than from sp646 or from the empty virus-like particle from sp79. We interpreted this observation to be, at least in part, because PfCSP-VLP expression in sp646 was half that of sp648 (expressions estimated at 0.7% and 1.4% of total soluble protein for sp646 and sp648, respectively). Based on these results, sp648 was selected for further testing in mice.

### Preparation of biomass and extract for mouse model studies

To generate material for inclusion in animal studies, sp648 was grown in a bioreactor, collected by centrifugation, washed, frozen at −80 °C, then lyophilized to generate dry spirulina powder. Total soluble protein was extracted from the powder by flash freezing in liquid nitrogen to lyse the cells, followed by centrifugation to collect the supernatant. Both the powder and extract fractions were analyzed by western blot to determine expression levels of the transgenic proteins. For sp648, PfCSP-VLP was estimated at 0.8% of the total biomass and 4% of the total soluble protein extract (Table 1). For administration in vaccine studies, dried powder was resuspended in HBSS with or without Montanide as indicated to a final concentration of 10 mg/mL. Extract concentration was batch dependent; hence, to avoid excessive handling during post-extraction processing, we used the most concentrated batch, at a final concentration of 8.7 mg/mL, in the vaccine trials. This translated to an inoculation dose of 15.7 µg following the addition of Montanide and 17.4 µg in cases where Montanide was not used. Sp79, with a similar expression profile estimated to be around 4% of total soluble protein, was administered at an extract concentration of 8.3 mg/mL.

**Table 1:** Vaccine dosages.

Delivery route	Composition of spirulina^+^	Final concentration of spirulina	Treatment volume per dose	Amount of spirulina per dose	Est. percent of vaccine construct	Dose of vaccine per treatment
Oral	Unpurified	50 mg/mL	200 µL	10 mg	0.8–1% of whole dried biomass	80–100 µg
Intranasal	Unpurified	50 mg/mL	50 µL	2.5 mg	0.8–1% of whole dried biomass	20–25 µg
Intranasal	Extract	7.8 mg/mL	50 µL	390 µg	4% of total soluble protein	15.7 µg* 17.4 µg**

^+^Unpurified = whole biomass powder resuspended in HBSS with 10% Montanide; Extract = total soluble protein in HBSS with 10% Montanide.

*Doses with* or without** Montanide.

### Intranasal and oral delivery of spirulina is well tolerated and immunogenic in mice

**Fig. 2:**
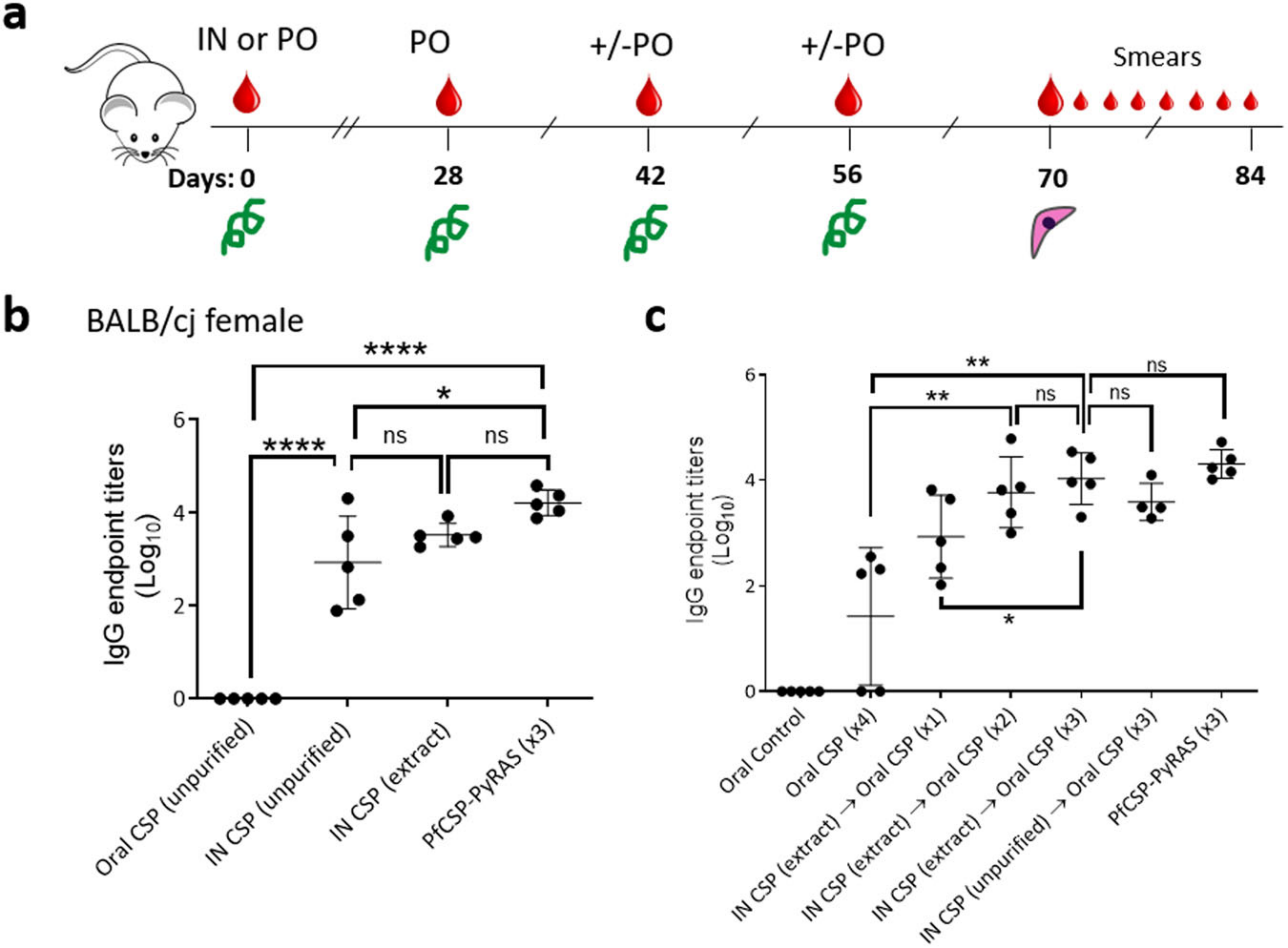
Immunogenicity following intranasal priming and oral boosting. **a** Study design. On Day 0, BALB/cJ mice were primed via one of three ways: 1. Orally (PO) with unpurified spirulina biomass resuspended in HBSS; 2. Intranasally (IN) with unpurified spirulina biomass resuspended in PBS; or 3. Intranasally with spirulina total soluble protein extract. All doses were supplemented with Montanide adjuvant. Oral Montanide-adjuvanted spirulina booster doses (containing control or PfCSP unpurified biomass) were then given 1–3 times on Days 28, 42, and up to 56. Subcutaneous challenge with 2 × 10^3^ wild-type PfCSP-expressing *P. yoelii* spz was conducted two weeks after the last booster. Serum was collected where indicated by large drops. Protection was assessed by blood smears (small drops). Endpoint titer ELISA results for PfCSP antibodies are shown two weeks after priming (**b**, Day 14) or two weeks after the third booster (**c**, Day 70). PfCSP-PyRAS (x3) refers to replicate wells of pooled sera obtained from female BALB/cJ mice immunized three times with irradiated Py spz expressing PfCSP as a benchmark control. Groups are as shown in the legend below each dataset; each data point is an individual mouse. Error bars show mean and standard deviation. *p < 0.05; **p < 0.01; ***p*<0.0001; ns not significant *p* > 0.05 (ANOVA with post-hoc Tukey test for multiple comparisons).

BALB/cJ mice were immunized by intranasal priming and oral boosting with spirulina expressing PfCSP-VLP (sp648) or empty-VLP (sp79) to assess vaccine tolerability and to measure antibody titers; these vaccines also contained the Montanide adjuvant at all doses (Fig. 2a). Two weeks after intranasal priming with sp648, female BALB/cJ mice mounted strong anti-PfCSP IgG responses, as measured by ELISA using an *E. coli* purified maltose binding protein (MBP)-NANP repeat fusion protein as capture antigen (Fig. 2b). Notably, the titers following only a single priming treatment approached that of mice immunized intravenously three times with recombinant PfCSP-expressing *P. yoelii* spz. Priming with the sp648 extract induced antibodies more consistently than priming with the unpurified sp648 product. Although the mean titers did not differ between these two groups, the range in response by individual mice primed intranasally with the unpurified biomass was considerably larger. Intranasally primed responses were significantly boosted by two or three oral doses of unpurified sp648, while a single oral booster after priming led to an intermediate level response (Fig. 2c). In addition to the systemic IgG responses, spirulina PfCSP vaccination also induced systemic IgA responses to NANP (Supplementary Fig. 2).

### Montanide adjuvant improves immunogenicity in male but not female mice

**Fig. 3:**
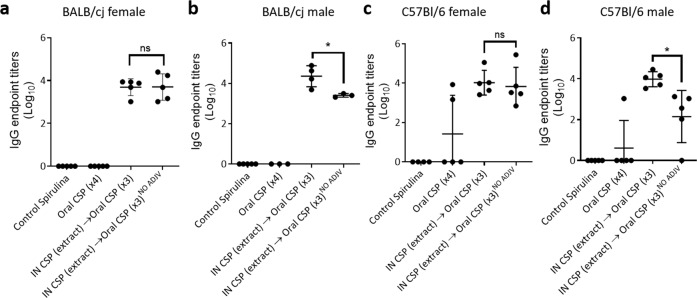
Immunogenicity in male and female mice with and without Montanide adjuvant. PfCSP ELISA titers in female BALB/cJ mice (**a**), male BALB/cJ mice (**b**), female C57Bl/6J mice (**c**), and male C57BL/6J mice (**d**) immunized as in Fig. 2 and assessed at the time of challenge (Day 70). Groups are as shown in the legend below each dataset; each data point is an individual mouse. NO ADJV indicates regimens where Montanide adjuvant was omitted at all priming and boosting steps. Error bars show mean and standard deviation. *p < 0.05; ns not significant *p* > 0.05 (ANOVA with post-hoc Tukey test for multiple comparisons).

To investigate whether the Montanide adjuvant was required, we compared the immunogenic intranasal sp648 extract priming and unpurified oral sp648 boosting regimen with and without Montanide in female and male BALB/cJ and C57Bl/6J mice. In female BALB/cJ mice, antibody titers were similar with and without Montanide (Fig. 3a). However, in male BALB/cJ mice, there was a statistically significant decrease in anti-PfCSP antibodies when Montanide was omitted (Fig. 3b). The same trends were observed in C57Bl/6 J mice with a statistically significant decrease in anti-PfCSP antibodies when Montanide was not used in male mice (Fig. 3c, d).

### Intranasal PfCSP-spirulina priming and oral boosting protects mice against spz challenge

**Fig. 4:**
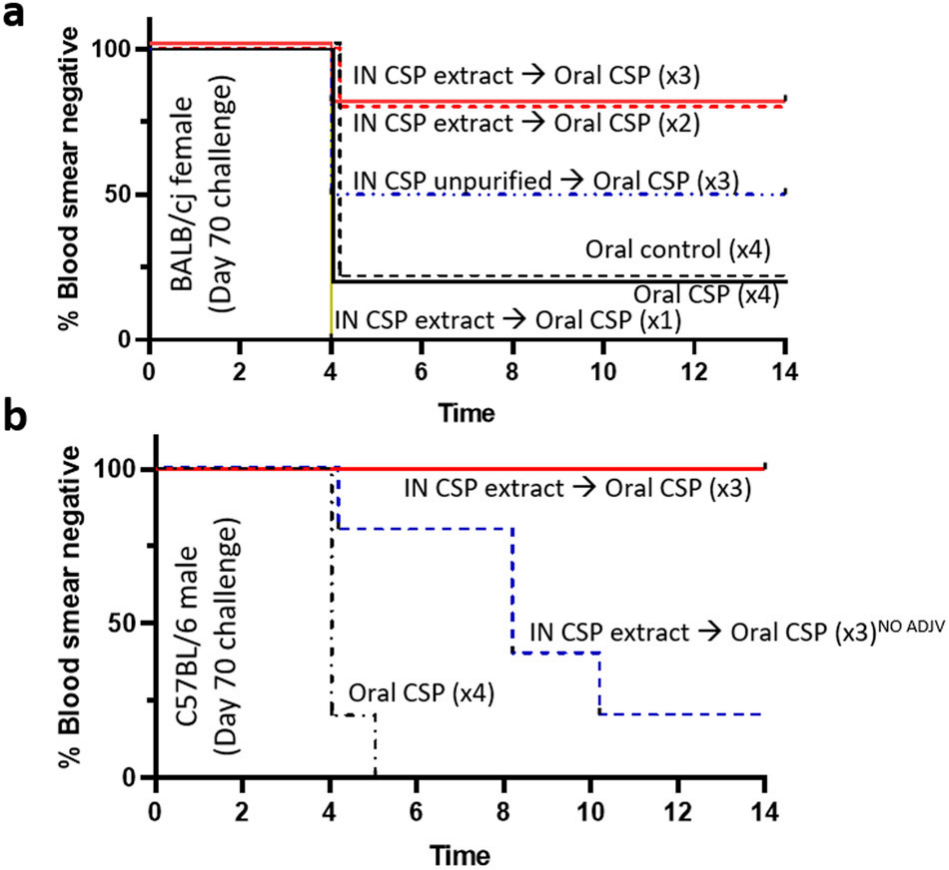
Protection after spirulina PfCSP vaccination. **a** Female BALB/cJ mice were intranasally primed and then orally boosted as in Fig. 2 and then challenged subcutaneously with PfCSP Py spz 14 days after the final vaccination *n* = 5 mice/group; representative of two replicate experiments. Survival curve shows endpoint of protection based on blood smear positivity. *p* = 0.01 (IN CSP extract priming and 2–3 doses of oral CSP vs. IN CSP extract priming and one dose of oral CSP), *p* = 0.07 (IN CSP extract priming and 2–3 doses oral CSP vs. oral control or oral CSP only), *p* = 0.37 (IN CSP unpurified priming and 3 doses of oral CSP vs. oral control or oral CSP only) (log-rank Mantel-Cox test). **b** Survival curve as in panel a for male C57Bl/6J mice. Vaccination regimens are shown in the text adjacent to each line. All vaccines shown contained Montanide except where indicated NO ADJV. *p* = 0.002 (adjuvanted vaccine vs. oral CSP only), *p* = 0.01 (no adjuvant vaccine vs. oral CSP only), *p* = 0.01 vaccines with vs. without adjuvant (log-rank Mantel-Cox test).

Female BALB/cJ and male C57Bl/6J mice immunized by intranasal priming and oral boosting as above were subjected to challenge with PfCSP-expressing *P. yoelii* spz (PfCSP-Py spz). Upon challenge, 80% of female BALB/cJ mice that received intranasal sp648 extract priming and two or three oral doses of sp648 were sterilely protected against challenge (Fig. 4a). Protection against challenge was also assessed in male C57Bl/6 mice on day 70 after vaccination with and without Montanide. Male C57Bl/6J mice vaccinated with the Montanide-containing intranasal priming and oral boosting regimen were completely protected against PfCSP *P. yoelii* spz challenge, whereas only 20% were protected after intranasal priming and oral boosting without Montanide (Fig. 4b).

**Fig. 5:**
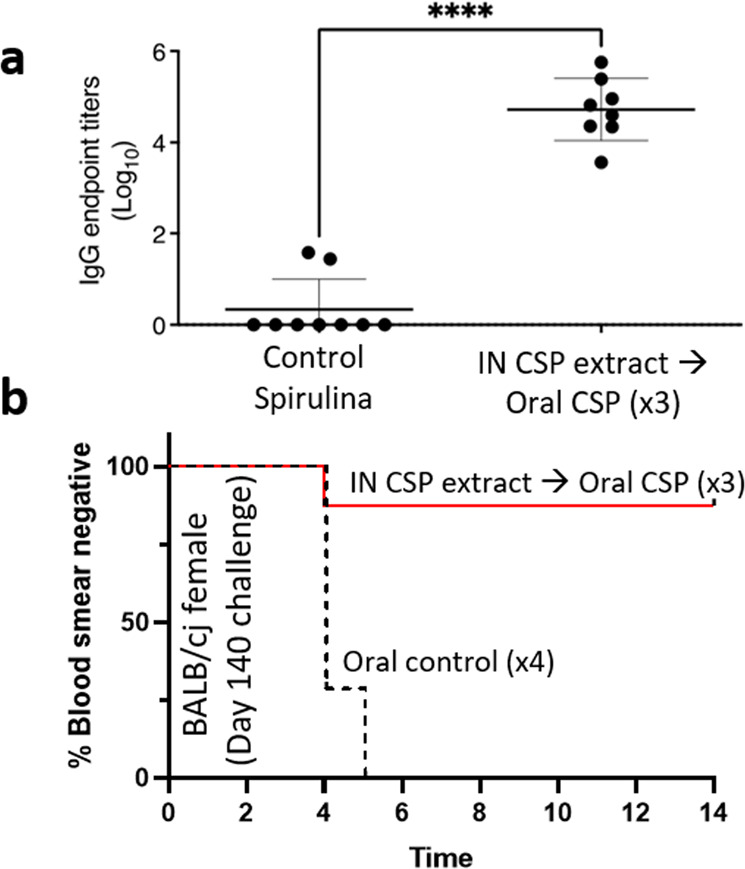
Durability of protection after spirulina PfCSP vaccination. Female Balb/cJ mice were primed (orally or intranasally as shown), then orally boosted, and then challenged intravenously with wild-type PfCSP Py spz 84 days after the last vaccination (Day 140). **a** Pre-challenge endpoint titer ELISA results for female BALB/cJ mice. Error bars show standard deviation; ****p < 0.0001 (log-rank Mantel-Cox test). Vaccinated datapoints represent titers of individual mice on day 139; controls datapoints are titers for pooled sera on each collection day throughout the trial since there was zero response detectable on day 139 in controls. **b** Survival curve of protection endpoint based on blood smear positivity. The vaccination regimen is shown in text adjacent to each line. *n* = 8 mice/group. All vaccines shown contained Montanide. IN intranasal. Survival curve *p* = 0.0008 (log-rank Mantel-Cox test).

To evaluate the durability of protection derived from the intranasal prime-oral boost regimen, BALB/cJ female mice were immunized intranasally with Montanide-adjuvanted sp648 extract, followed by oral boosting (3×) with Montanide-adjuvanted sp648 biomass. Figure 5a shows the mean antibody titers for these mice on day 139 compared to the titers in control mice vaccinated with the empty VLP. Control titers consistently below the calculated cutoff based on naïve sera. Titers in the sp648 vaccinated mice (mean 4.723, SD 0.6782) were not statistically different from those seen at pre-challenge time points in similarly vaccinated mice shown in Fig. 2c that were challenged at shorter post-vaccination intervals (mean 4.03, SD 0.49). Indeed, antibody titers to NANP as well as against the empty VLP changed little over time between days 56 and 139 (Supplementary Fig. 3). When challenged three months post-immunization, 87.5% of the intranasally primed and orally boosted mice were sterilely protected (Fig. 5b).

**Fig. 6:**
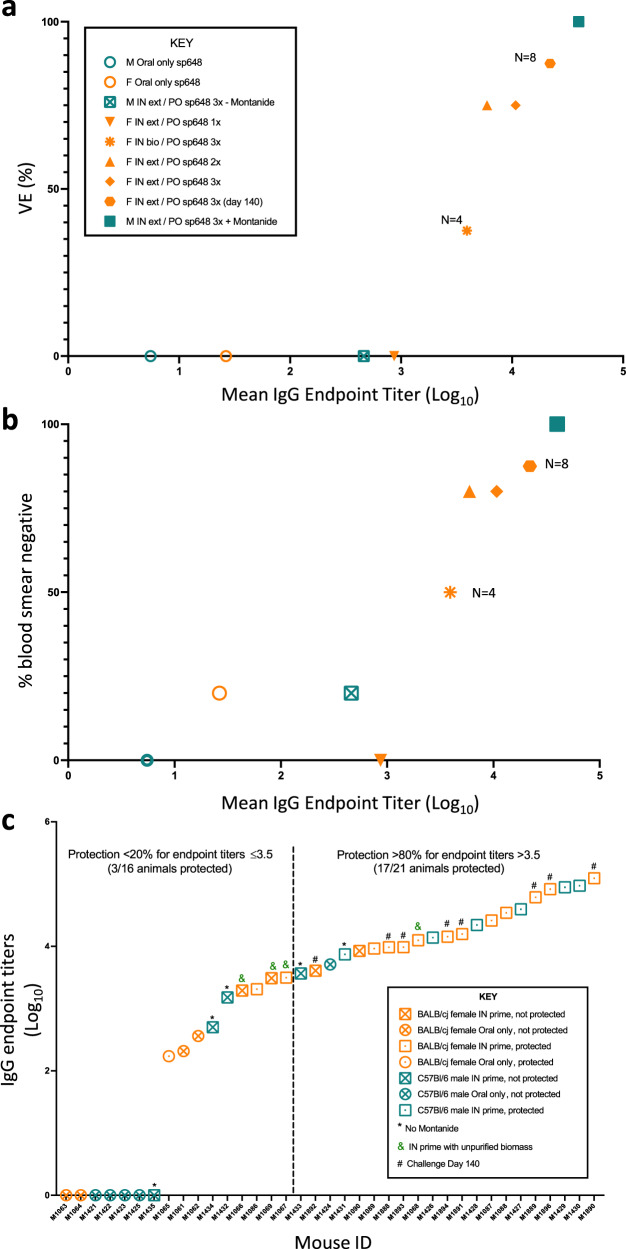
Relationship between pre-challenge anti-PfCSP antibody titers and protection from challenge. **a** Vaccine Efficacy (VE) was calculated for each group as 1 – risk ratio (×100) and plotted against respective mean endpoint titer. **b** The percentage of mice that were blood smear negative post-challenge was plotted against the respective mean NANP-specific IgG endpoint titer at the time of challenge. For A and B for all groups except where indicated, challenge took place on day 70 of the respective trial. In **a**–**b**, each data point represents a group of mice as shown in the inset key (*n* = 5 mice/group unless otherwise indicated). **c** Individual mouse NANP-specific IgG endpoint titers at the time of challenge were plotted compared to the challenge outcomes. Each data point represents an individual mouse with the corresponding groups identified in the inset key.

To evaluate potential correlates of protection, we compared the results of our challenge experiments to the estimated IgG endpoint titers for NANP by group mean as well as by individual mice. Higher mean anti-NANP IgG titers were associated with higher VE (Fig. 6a) and more blood smear negative results (Fig. 6b). Plotting individual mice by increasing endpoint titers, we estimated a threshold of protection of at least 80% when the IgG endpoint titer was >3.5 (Fig. 6c).

## Discussion

Current malaria vaccines like RTS,S and R21 demonstrate that the generation of spz invasion blocking antibodies is effective at preventing disease. However, these needle-based vaccines present logistical challenges in resource-limited settings. Here, we describe the use of an algae-based expression platform that can be engineered to generate a shelf-stable, effective malaria vaccine for non-parenteral delivery. Specifically, we show that intranasal priming with PfCSP-bearing spirulina generates a strong IgG response that can be boosted and sustained by oral consumption of unpurified spirulina biomass expressing PfCSP. The addition of a Montanide adjuvant also elevated responses in male mice to that observed in female mice without adjuvant. Most importantly, these antibodies protected mice against spz challenge. This successful use of a spirulina-based malaria vaccine prompts further development of this platform in vaccine research against malaria and other pathogens of public health importance.

We have engineered spirulina to express a variety of exogenous antigens such as the acid-stable VLPs bearing PfCSP described here. VLPs are non-infectious, robust, and highly immunogenic nanoparticles that spontaneously form when viral capsid proteins are expressed in heterologous systems. VLPs have been established as an effective vaccine delivery system and are used in the injected RTS,S vaccine as well as several veterinary vaccines. In addition, orally delivered VLPs have been shown to be safe and effective in healthy human volunteers^31–33^. Of the many variations of VLP-based vaccine constructs that we have generated in spirulina, we have observed expression levels up to 6% of soluble protein. Other spirulina strains, particularly those expressing heavy chain-only antibodies, have reached expression levels of ~15% of total biomass^16^. Compared to plant-based vaccines, which also offer a needle-free alternative to traditional immunization^34^, expression of heterologous proteins in spirulina is 10- to 100-fold higher. Moreover, our data here and elsewhere^16^ demonstrate that spirulina expressed constructs retain stability and bioactivity in the absence of cold-chain storage, allowing for the stockpiling of material in remote and resource-limited settings. If a Montanide gel adjuvant is required, adjuvant stability will also need to be evaluated. The manufacturer (Seppic) reports that the Montanide used herein is stable for more than one year at 4 °C, for one year at 20 °C, and for one month at 37 °C.

There are relatively few examples of intranasal vaccines for systemic and/or blood-based pathogens. For malaria, a baculovirus-based vaccine was administered as an intranasal liquid that induced anti-MSP1 antibodies in mice and achieved protection^35^. Similarly, intranasal immunization of mice with Pfs25 complexed with a cholera toxin adjuvant-induced antibodies with in vitro transmission-blocking activity^36^. Intranasal administration also enhanced the immunogenicity of SPf66-loaded vaccine microparticles in BALB/c mice^37^. Efforts were made to use *Lactococcus* as an intranasal *Plasmodium* vaccine, but undesirable age-dependent changes possibly arising from oral tolerance to *Lactobacillus* were observed^38,39^. Other studies done using intranasally dosed PfCSP coupled to flagellin as an adjuvant showed that this vaccine was actively taken up in the Nasal Associated Lymphoid Tissue (NALT)^40^, which we hypothesize is similarly responsible for the immunogenicity of intranasal immunization seen for our spirulina-based vaccine and will be the subject of future research.

Depending on the formulation, oral vaccines can activate cellular and humoral immunity including serum IgG that can protect against both mucosally invasive pathogens and systemic pathogens^41^. Such vaccines have shown promise for malaria, HBV, and JEV^42^. Many different oral delivery vehicles have been used including recombinant proteins^43^, microparticles^44,45^, *Salmonella*^46–52^, lactococci^38,53,54^, and non-spirulina algae like *Chlamydomonas*^55^. However, most of these systems require extensive purification or use of live vectors for antigen delivery. Studies in the alga *Chlamydomonas reinhardtii* are most closely related to the spirulina-based platform described here. *C. reinhardtii* was used to express blood-stage malarial proteins that induced protective antibody responses when purified and admixed with recombinant heat-labile toxin (homologous to cholera toxin B (CTB)) and fed to mice^56^. In another study, a Pfs25-CTB fusion expressed in *C. reinhardtii* was protective in transmission blocking studies when injected intraperitoneally^55^, but was not protective when administered orally as non-purified biomass (IgA but not IgG was induced)^57^. The low expression (0.09% of total soluble protein) and/or degradation in the stomach were invoked as possible explanations for its failure. Since oral PfCSP spirulina boosted intranasally primed responses, it is tempting to speculate that oral PfCSP spirulina might be able to similarly boost antibody responses initially primed by RTS,S and/or R21 vaccines, and this approach could also be tested in future studies.

There are several limitations to this study that will be explored in future efforts. First, the PfCSP sp648 construct tested here did not contain the PfCSP N- and/or C-terminal domains that contain known T-cell helper epitopes^58^. Given the strong antibody responses observed here, it is likely that T cell help was instead derived from CD4^+^ T cell responses to the WHcAg VLP since specific T-helper cell responses are known to be induced by Woodchuck hepatitis virus infection against the WHcAg^59–61^. Future studies may be warranted to test additional versions of this vaccine that include some of the known and proposed N- and C-terminal T cell helper epitopes. Second, only inbred mice were studied; future efforts should test vaccine performance in outbred mice and/or non-human primates to continue translational development of this vaccine candidate. Third, most of the studies conducted herein utilized the Montanide adjuvant for the oral booster doses. We observed that this adjuvant increased responses in male mice, but was dispensable in female mice with respect to immunogenicity. Sex-specific differences in vaccine-induced immune responses are increasingly recognized^62,63^ including differences in *Plasmodium* sporozoite immunization in mice^64^. In general, females trended toward higher antibody responses and more frequent adverse reactions than males^65^. Our findings warrant future studies with and without adjuvant as part of candidate vaccine development, as it would simplify production if the vaccine can achieve protection alone.

Fourth, the challenge model here used a needle and syringe to inject wild-type spz into the subcutaneous space. Future studies should consider testing this vaccine against mosquito bite challenge in mice and/or non-human primates. Fifth, intranasal vaccination of mice and humans face anatomical and immunological differences^66,67^, which will need to be explored in subsequent studies. Finally, the NANP target region of PfCSP is not the only region of CSP that has shown promise for spz invasion blocking. There is evidence that the immunodominant NANP repeats may reduce the formation of neutralizing antibodies to other sub-dominant epitopes^68^. Thus, there may be value in adding or using other parts of PfCSP in a vaccine, and some of these modifications are already underway.

Despite progress made in recent decades, malaria still claims over half a million lives every year, primarily children under five. Malaria is a preventable and treatable disease, yet healthcare systems in endemic areas often have limited resources. Vaccines and other medicines that rely on cold chain delivery and/or require needle-based administration may be slow to reach those in need. For example, WHO has indicated that “in countries where cold chain capacity is already limited/exceeded, the introduction of RTS,S vaccine may pose a logistics challenge”^69^. Supply chain bottlenecks have been similarly highlighted during the COVID-19 pandemic, for example, periodic shortages in syringes required for COVID-19 vaccine administration. Non-parenteral delivery of temperature-stable spirulina-based vaccines and therapeutics represents an opportunity to address some of the specific challenges faced in resource-limited settings and may help fill access gaps that perpetuate cycles of poverty and poor health in these regions.

## Methods

### Mouse resources and ethics

BALB/cJ and C57Bl/6J (4–6 weeks old, female and male) were obtained from Jackson Laboratories. All animal procedures were conducted in accordance with and approved by the University of Washington Institutional Animal Care and Use Committee under protocol 4317-01, which adheres to the NIH Office of Laboratory Animal Welfare standards.

### Preparation of sporozoites

The PfCSP-expressing *P. yoelii* strain was kindly donated by Moriya Tsuji (Columbia University). Spz-infected mosquitoes were reared at Seattle Children’s Research Institute Insectary. Spz were isolated from salivary glands 14–16 days post-infection by dissection in Schneider’s insect medium and purified by centrifugation in a 17% w/v filter-sterilized Accudenz gradient^70^. Briefly, dissected spz were overlaid onto the Accudenz cushion, and the tube was then centrifuged at 2500 × *g* at room temperature for 20 min (without brake). Spz in the interface were collected, pelleted, resuspended in Schneider’s medium, and counted on a hemocytometer.

### Preparation of spirulina biomass and extract

Cultures of NIES39 strain spirulina were transformed^16^. Briefly, cultures were grown to an optical density (OD700 mL^−1^) 0.5–1.0, then harvested by centrifugation for 10 min at 1600 × *g*. Cells were then washed and resuspended in Spirulina-Ogawa-Terui (SOT) algal culture media (ref. ^71^; made in-house at Lumen) followed by 3 h incubation at room temperature with plasmid DNA at 10 ng/µL final concentration. Samples were then transferred to tubes containing fresh SOT media and incubated over night at 25–30 °C under 50–100 µEi of fluorescent light. Additional SOT containing appropriate selective antibiotics was then added to begin selection. To prepare material for animal trials, strains were grown in SOT supplemented with 2.5–5 µg/mL of streptomycin in Multitron incubators at 35 °C, 0.5% CO_2_, 110–150 µEi of light, and shaking at 120–200 rpm. For large-scale harvests, biomass was collected and washed three times in deionized water by centrifugation. Pellets were frozen in −80 °C and lyophilized to generate dried biomass. For biomass that was processed into dry powder via spray drying, cultures were filter washed over mesh screens with a 0.2% solution of trehalose in water, then dried in a Buchi B290 spray dryer set to 142 °C inlet temperature and 60 °C outlet temperature. Inlet temperature was modulated to obtain target outlet temperature. To make extract, dried biomass was brought to 50 mg/mL in Phosphate Buffered Saline (PBS) supplemented with protease inhibitor tablets (Pierce), and subject to three rounds of flash freezing in liquid nitrogen, with intermediate steps at 37 °C, followed by centrifugation at 18,000 × *g* for 20 min to collect supernatant. To verify protein expression, samples were run on SDS-PAGE followed by western blot to visualize the His-tagged (sp648) or Myc-tagged (sp79) proteins.

### Sucrose density gradient studies

To prepare crude lysate for sucrose gradients, mature spirulina cultures were washed in TBS and resuspended in 25 mM Tris at pH 7.4 and kept on ice. Samples were sonicated at 20% amplitude four times for 30 seconds, with 15 s pauses, using the Qsonica Q700. Sucrose gradient solutions were made in 1× PBS at the following percentages: 70%, 60%, 50%, 40%, 30%, and 20%. A density gradient was made in 14 mm × 89 mm Beckman-Coulter centrifuge tubes by pipetting sucrose solutions on top of one another in decreasing order, with the highest concentrations at the bottom. Spirulina extracts were then layered on the top gradient. Samples were centrifuged at 115,000 × *g* for 16 h at 4 °C in a Beckman-Coulter Optima L-80 XP Ultra Centrifuge with swinging bucket rotor. An 18-gauge needle was used to puncture the bottom of the tubes to collect fractions in sequential 1 mL aliquots into Eppendorf tubes for subsequent analysis by western blot. Source data files of unprocessed gel and western blot images are available in the online version.

### Long-term storage analysis by SDS-PAGE western blot and automated immunoblot

Aliquots of lyophilized sp79 and sp82 biomass were stored in sealed moisture-free chambers away from light for one year at −80 °C, −20 °C, 4 °C, 25 °C, 37 °C and 42 °C. Samples were brought to 1 mg/mL in SDS sample buffer, boiled for 5 min at 100 °C, and stored in −80 °C until analysis by SDS-PAGE followed by western blot with rabbit anti-Myc antibody (Rockland) at 1:3000 and donkey anti-rabbit (Invitrogen) at 1:10,000. ImageJ software was used to estimate band intensity relative to a sample taken to represent time zero (from fresh harvest). For sp648, biomass samples were processed and stored under identical conditions as above for 10 months. To harvest the total soluble protein, measured aliquots were resuspended in PBS with Halt Protease Inhibitor and lysed on a Precellys Evolution Homogenizer (Bertin Instruments) using Lysis Matrix B (MP Bio) beads, followed by centrifugation to collect the supernatant. Automated immunoblotting was done via the Jess^TM^ Simple Western nano-immunoassay system (ProteinSimple, San Jose, CA, USA), following the manufacturer’s protocol, using mouse anti-His (ThermoFisher Scientific) followed by goat anti-mouse HRP conjugate (ProteinSimple) and rabbit-NANP (Alpha Diagnostic) followed by goat anti-rabbit HRP conjugate (ProteinSimple).

### Immunization studies

Groups of 4–8 mice per group were primed intranasally or orally prior to receiving three oral boosts in two-week intervals starting four weeks after the initial prime. Intranasal immunization was done by gradually inoculating 50 µL volumes of either 2.5 mg of spirulina resuspended in Hank’s balanced salt solution (HBSS) (~20 µg of vaccine construct) or its purified extract (15–20 µg of VLP) to isoflurane-anesthetized mice. For oral immunization, mice were fasted for four hours prior to and two hours after oral gavage administration of 10 mg spirulina (~80 µg of vaccine construct) resuspended in 200 µL HBSS. All vaccines except those labeled as “No Adjuvant” contained 10% Montanide (Seppic Montanide gel 01 PR #36067D) added to the vaccine formulations at the time of intranasal and oral administrations.

For spz immunizations, 2 × 10^4^ radiation-attenuated PfCSP-Py spz resuspended in 150 µL Schneider’s insect medium were administered three times at 28-day intervals by retro-orbital intravenous injection in isoflurane-anesthetized BALB/cJ female mice.

### ELISA immunoassays

Samples were collected from mice at baseline, prior to each dose of immunization at two-week intervals and prior to challenge. Briefly, 100–150 µL blood was collected by submental bleeds and allowed to coagulate for up to two hours at room temperature and then centrifuged at 4 °C at 2000 × *g* for 10 min to isolate serum. Serum was stored in −20 °C until analyzed.

For ELISAs, 96-well high binding plates (Greiner Cat-No 655085) were coated with 50 µg per well of spirulina extract or with 500 ng per well of purified target antigen, either MBP-NANP, empty VLP, or MBP alone as a negative control, and incubated overnight at 4 °C in sealed chambers. Plates were then washed 3× in PBS with 0.05% Tween-20 (PBS-T) before blocking in PBS-T with 5% non-fat milk (PBS-T-NFM) for 2 h at room temperature. Samples were set up in duplicate with three-fold serial dilutions in PBS-T-NFM starting at 1:100 on separate low binding plates then transferred to the corresponding well on antigen-coated plates and incubated overnight at 4 °C in a sealed chamber. Plates were then washed three times in PBS-T and incubated with HRP conjugated goat anti-mouse IgG (Fisher Cat-No 31430) at 1:5000 dilution for 1 h, followed by three washes in PBS-T and one wash in PBS. Plates were developed using One-step Ultra TMB-ELISA from Thermo Scientific (product number 34029) and stopped with 2.5 M sulfuric acid. Plates were read on a SpectraMax M3 microplate reader from Molecular Devices. Analysis was done using Microsoft Excel and Prism (Graph Pad) software. Naïve sera (day 0) response to antigen was used to calculate cutoff levels based on published methods^72^ using the 99% confidence level standard deviation multiplier for the N value of the respective assay.

### Challenge studies

Immunized mice and relevant controls were challenged with 2 × 10^3^ purified PfCSP-Py spz by subcutaneous injection in the pouch between the hind leg and abdomen two weeks or three months after the final boost. Mice were monitored for breakthrough infection by Giemsa staining of blood smears 3–14 days post-challenge.

### Statistical analysis

Statistical tests are indicated in figure legends and were conducted using Prism (GraphPad). For ELISAs, endpoint titers were estimated using a four-parameter logistic model on each dilution curve and analysis of variance (ANOVA) was used to compare means amongst groups, followed by post-hoc Tukey tests for multiple comparisons. For survival analyses, log-rank Mantel-Cox tests were used. Significant results are indicated in figures with relevant *p* values defined in each figure legend. Comparisons not indicated in figures or figure legends were non-significant with *p* > 0.05.

### Reporting summary

Further information on research design is available in the Nature Research Reporting Summary linked to this article.

## Supplementary information

Supplementary Information

REPORTING SUMMARY

## Data Availability

The authors declare that the data supporting the findings of this study are available within the main and supplemental figures. Data (i.e., ELISA measurements, daily qualitative blood smear results) are available by emailing the corresponding author (S.C.M.).
