# Supplementary material for: Needle-free, spirulina-produced Plasmodium falciparum circumsporozoite vaccination provides sterile protection against pre-erythrocytic malaria in mice
Source: NPJ Vaccines. 2022 Oct 4;7:113. doi: 10.1038/s41541-022-00534-5 (PMC9532447; doi:10.1038/s41541-022-00534-5)
Supplement: Supplementary file 1 — Supplementary Information [file 41541_2022_534_MOESM1_ESM.pdf]

Supplementary Information for "A needle-free, mucosally-delivered spirulina platform *Plasmodium falciparum* circumsporozoite vaccine provides sterile protection against pre-erythrocytic malaria in mice" (Saveria, Parthiban, et al.)

Supplementary Figure 1. Stability testing of spirulina strains sp82 (PyCSP-expressing strain) and sp648 (PfCSP-expressing strain). a Samples of lyophilized sp82 biomass were stored in duplicate at 42°C, 37°C, 25°C, 4°C, -20°C, and -80°C for one year. Samples were then separated by reducing SDS-PAGE followed by western blotting. Rabbit anti-Myc antibody was used at 1:3,000 for the detection of C-terminal Myc tag, followed by anti-rabbit HRP. Percent intensity relative to day zero is shown in each lane. Rep, replicate. **b** and **c** Samples of lyophilized (b) or spray dried (c) sp648 were stored for 10 months under the same conditions as above. Soluble protein expression levels were then measured by the Protein Simple Jess western blot platform using antibodies to both the C-terminal His tag as well as the PfCSP NANP repeat. Accompanying tables list expression of the PfCSP-VLP construct as a percent of total spirulina dry weight measured in the respective sample. (*sample lost during analysis). Positions of molecular weight markers are shown in kDa.

a

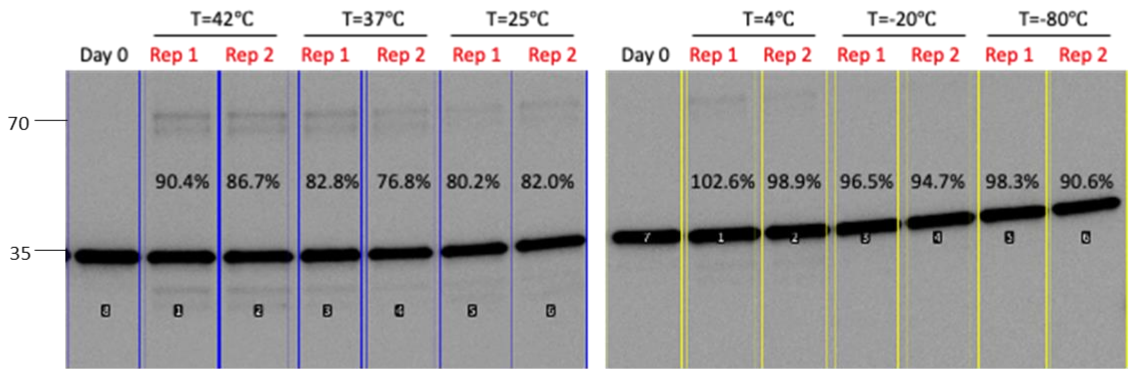

b

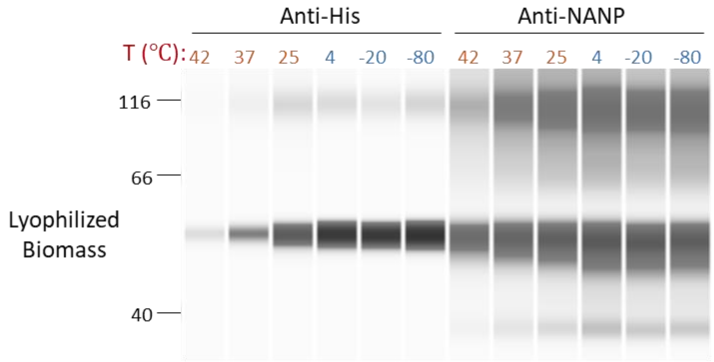

Temp (Celsius)	Percent Expression (of total dry weight)	
	Anti-His	Anti-NANP
42	0.75	0.54
37	1.16	0.81
25	1.96	1.05
4	2.23	1.58
-20	2.11	1.26
-80	2.83	1.1

c

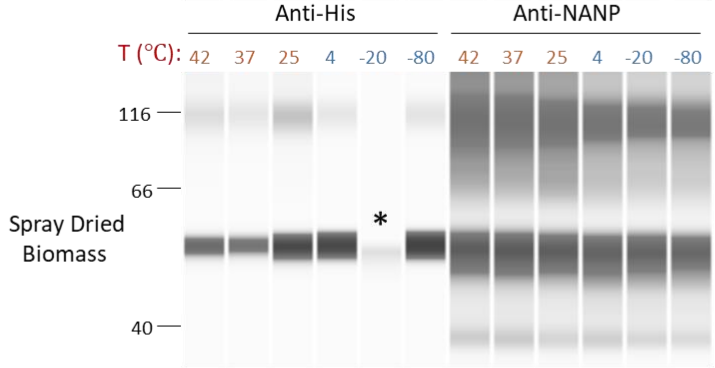

Temp (Celsius)	Percent Expression (of total dry weight)	
	Anti-His	Anti-NANP
42	1.55	1.4
37	1.38	1.63
25	2.43	1.22
4	2.57	2.21
-20	0.69	1.77
-80	3.15	1.33

Supplementary Figure 2. IgA endpoint titers for PfCSP following spirulina PfCSP vaccination. Endpoint titer ELISA results are shown two weeks after the third booster prior to challenge (Day 70). Error bars show standard deviation.

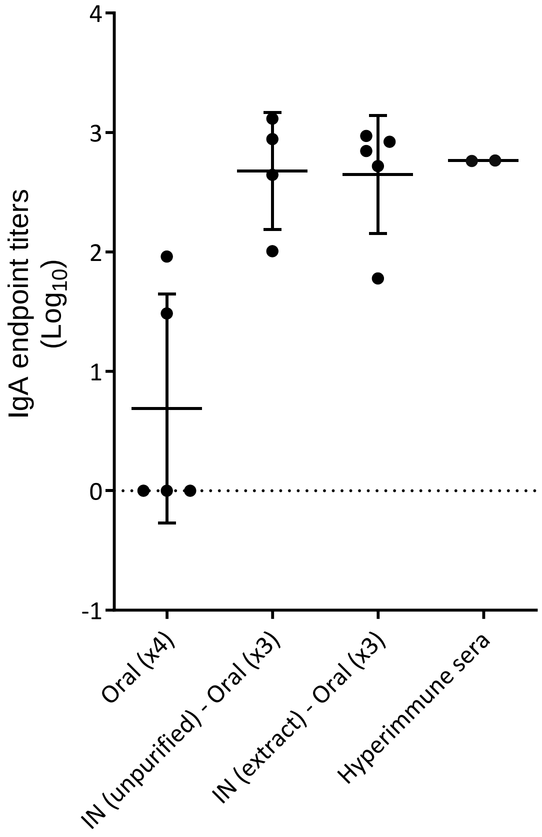

Supplementary Figure 3. Longevity of responses to NANP and to the empty VLP in spirulina vaccination. Mice vaccinated with PfCSP-VLP mounted persistent antibody responses to both NANP (**a**) and the empty VLP (**b**). Error bars show standard deviation. N=8/day; one sample from Day 98 was lost.

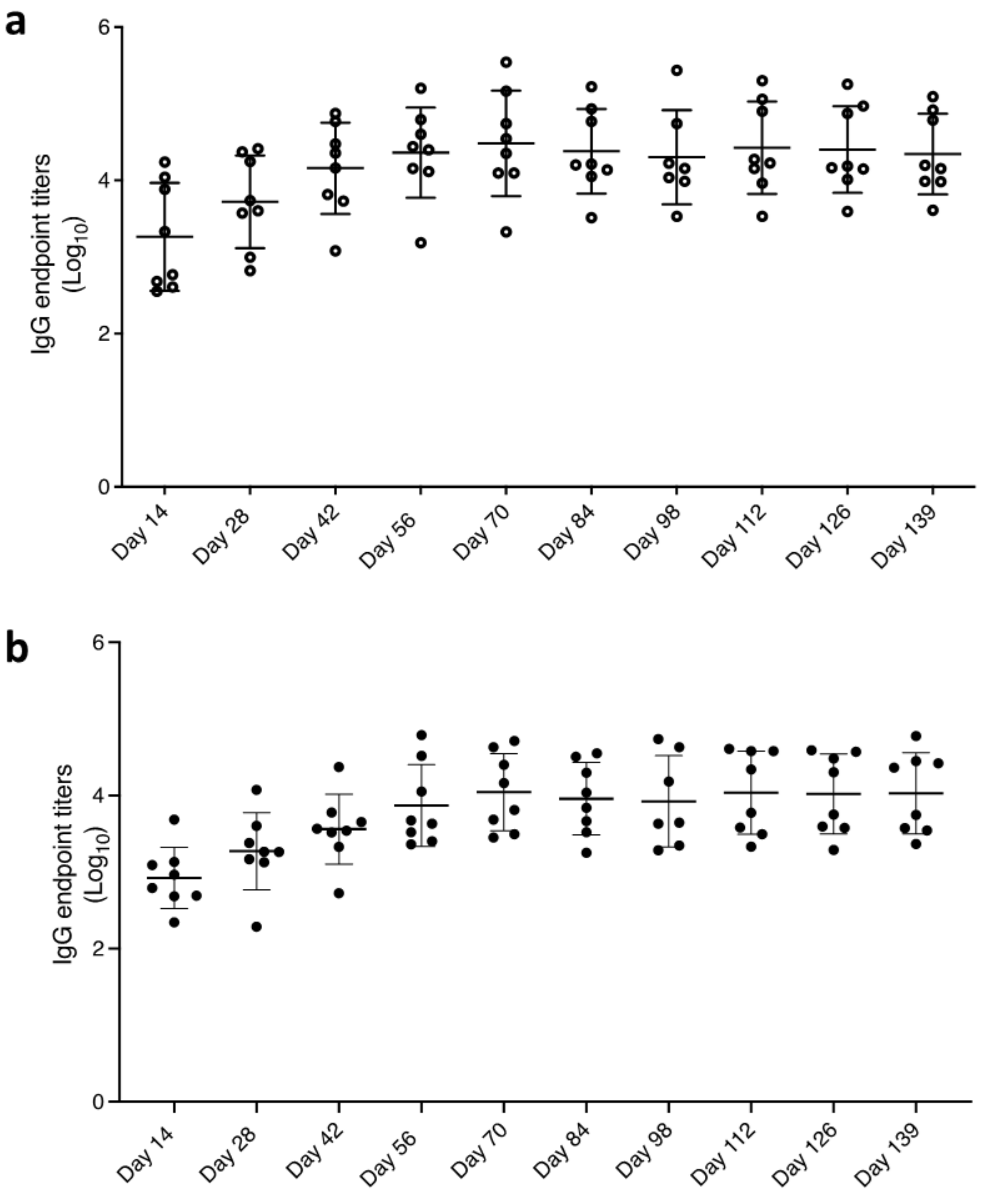

Source Data File - Western blots in Figure 1e in "A needle-free, mucosally-delivered spirulina platform *Plasmodium falciparum* circumsporozoite vaccine provides sterile protection against pre-erythrocytic malaria in mice" by Saveria, Parthiban, et al.

One-mL fractions were collected by bottom puncture of the tube shown in Figures 1d-e with every other fraction resolved by SDS-PAGE and Western blotting using anti-Myc-HRP. The image on the left is the uncropped anti-Myc Western blots for sucrose gradients of sp82 with no SDS pre-treatment (top) or with SDS pre-treatment to disrupt particles (bottom). The image on the right is the digitized gel showing the molecular weight markers for the corresponding Western blots. The molecular weight marker was PageRuler Plus Prestained Protein Ladder (ThermoFisher).

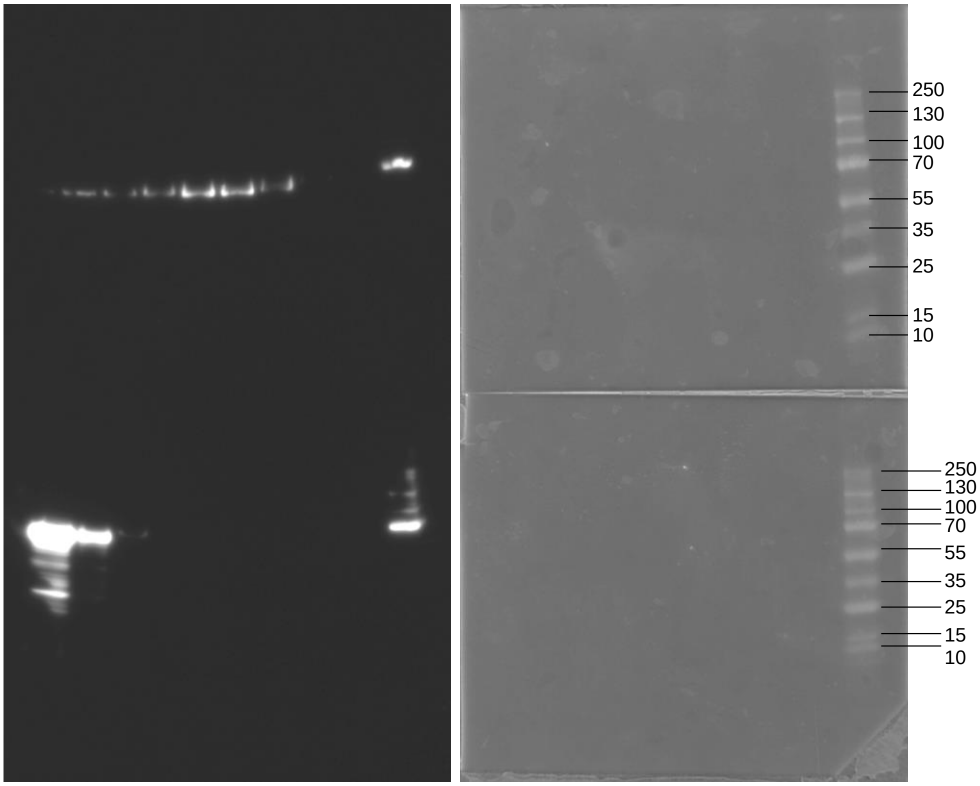

Source Data File – Western blots in Supplementary Figure 1a in “A needle-free, mucosally-delivered spirulina platform *Plasmodium falciparum* circumsporozoite vaccine provides sterile protection against pre-erythrocytic malaria in mice” by Saveria, Parthiban, et al.

TIFF images of raw data for sp82 long term stability. Two gels were run back-to-back in the same electrophoresis chamber, with all subsequent incubations done at the same time. Western blot development took place simultaneously and images were captured using the GE ImageQuant LAS4000.

Left side: digitized gel capture
Right side: Western blot signal capture

The top portion of each image (from left to right) shows: Ladder, Day 0 (in duplicate), T=42°C (in duplicate), T=37°C (in duplicate), T=25°C (in duplicate), and purified empty VLP.

The bottom portion of each image (from left to right) shows: Ladder, Day 0 (in duplicate), T=4°C (in duplicate), T=-20°C (in duplicate), T=-80°C (in duplicate), and purified empty VLP.

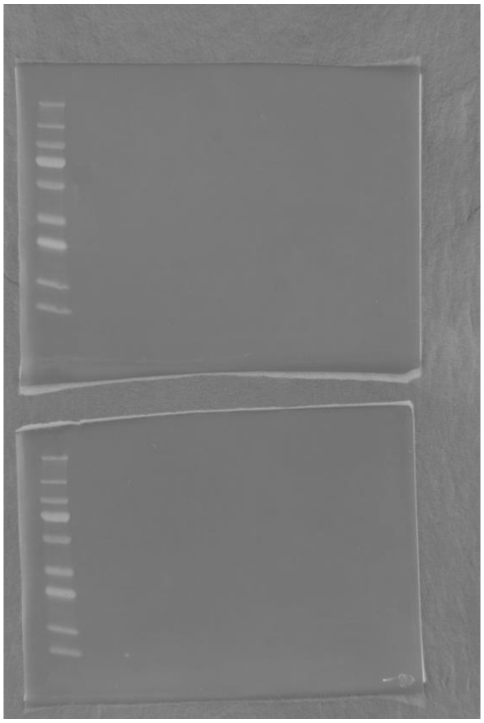

Gel image

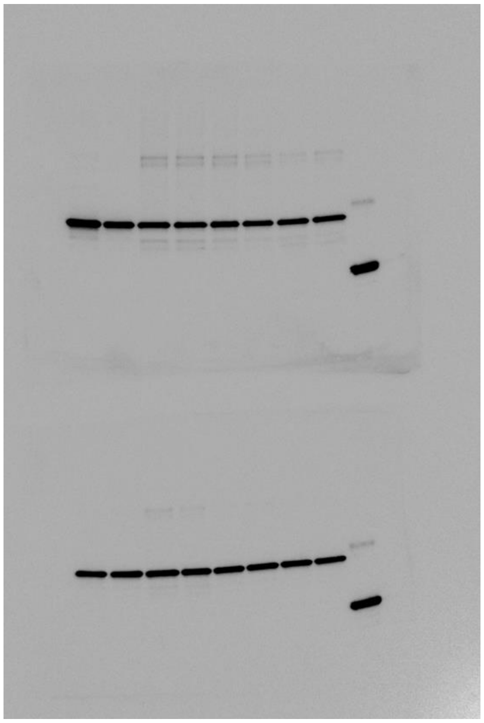

Western blot image
