# Supplementary material for: Needle-free, spirulina-produced Plasmodium falciparum circumsporozoite vaccination provides sterile protection against pre-erythrocytic malaria in mice
Source: NPJ Vaccines. 2022 Oct 4;7:113. doi: 10.1038/s41541-022-00534-5 (PMC9532447; doi:10.1038/s41541-022-00534-5)
Supplement: Supplementary file 2 — REPORTING SUMMARY [file 41541_2022_534_MOESM2_ESM.pdf]

nature portfolio

Corresponding author(s): Michael Tasch, Sean C. Murphy

Last updated by author(s): Aug 11, 2022

Reporting Summary

Nature Portfolio wishes to improve the reproducibility of the work that we publish. This form provides structure for consistency and transparency in reporting. For further information on Nature Portfolio policies, see our Editorial Policies and the Editorial Policy Checklist.

Statistics

For all statistical analyses, confirm that the following items are present in the figure legend, table legend, main text, or Methods section.

n/a	Confirmed	
☐	☒	The exact sample size (n) for each experimental group/condition, given as a discrete number and unit of measurement
☐	☒	A statement on whether measurements were taken from distinct samples or whether the same sample was measured repeatedly
☐	☒	The statistical test(s) used AND whether they are one- or two-sided<br>*Only common tests should be described solely by name; describe more complex techniques in the Methods section.*
☒	☐	A description of all covariates tested
☐	☒	A description of any assumptions or corrections, such as tests of normality and adjustment for multiple comparisons
☐	☒	A full description of the statistical parameters including central tendency (e.g. means) or other basic estimates (e.g. regression coefficient) AND variation (e.g. standard deviation) or associated estimates of uncertainty (e.g. confidence intervals)
☐	☒	For null hypothesis testing, the test statistic (e.g. F, t, r) with confidence intervals, effect sizes, degrees of freedom and P value noted<br>*Give P values as exact values whenever suitable.*
☒	☐	For Bayesian analysis, information on the choice of priors and Markov chain Monte Carlo settings
☒	☐	For hierarchical and complex designs, identification of the appropriate level for tests and full reporting of outcomes
☒	☐	Estimates of effect sizes (e.g. Cohen's d, Pearson's r), indicating how they were calculated

Our web collection on statistics for biologists contains articles on many of the points above.

Software and code

Policy information about availability of computer code

Data collection	Analysis was Plates were read on SpectraMax M3 microplate reader<br>ELISA plates were read on a SpectraMax M3 microplate reader from Molecular Devices.
Data analysis	Analysis was done using Microsoft Excel and Prism (Graph Pad) software.

For manuscripts utilizing custom algorithms or software that are central to the research but not yet described in published literature, software must be made available to editors and reviewers. We strongly encourage code deposition in a community repository (e.g. GitHub). See the Nature Portfolio guidelines for submitting code & software for further information.

Data

Policy information about availability of data

All manuscripts must include a data availability statement. This statement should provide the following information, where applicable:

- Accession codes, unique identifiers, or web links for publicly available datasets
- A description of any restrictions on data availability
- For clinical datasets or third party data, please ensure that the statement adheres to our policy

The datasets generated during the study are included in the published article. Two source files are provided in the online version of the manuscript. Additional details are available from the corresponding author on reasonable request.

Human research participants

Policy information about studies involving human research participants and Sex and Gender in Research.

Reporting on sex and gender	N/A
Population characteristics	N/A
Recruitment	N/A
Ethics oversight	N/A

Note that full information on the approval of the study protocol must also be provided in the manuscript.

Field-specific reporting

Please select the one below that is the best fit for your research. If you are not sure, read the appropriate sections before making your selection.

☒ Life sciences ☐ Behavioural & social sciences ☐ Ecological, evolutionary & environmental sciences

For a reference copy of the document with all sections, see nature.com/documents/nr-reporting-summary-flat.pdf

Life sciences study design

All studies must disclose on these points even when the disclosure is negative.

Sample size	Power calculations determined that n=5 mice per group for ELISA endpoints have 90% power to show a two-fold difference in titers between groups with expected 25% assay variability in response magnitude.
Data exclusions	None.
Replication	Immunogenicity studies were repeated twice per strain. Short-term challenge experiments were performed twice as well. The durability challenge experiment was performed once with a larger number of mice.
Randomization	Not relevant to this study. Animals were assigned to groups and the inert vaccine was administered. Note that the times and conditions of wild-type live sporozoite challenge were carefully recorded in order to ensure that there was no temporal relationship between the animals challenged first vs. last on a given challenge day.
Blinding	Investigators were not blinded to study groups.

Reporting for specific materials, systems and methods

We require information from authors about some types of materials, experimental systems and methods used in many studies. Here, indicate whether each material, system or method listed is relevant to your study. If you are not sure if a list item applies to your research, read the appropriate section before selecting a response.

Materials & experimental systems

n/a	Involved in the study	
☐	☒	Antibodies
☒	☐	Eukaryotic cell lines
☒	☐	Palaeontology and archaeology
☐	☒	Animals and other organisms
☒	☐	Clinical data
☒	☐	Dual use research of concern

Methods

n/a	Involved in the study	
☒	☐	ChIP-seq
☒	☐	Flow cytometry
☒	☐	MRI-based neuroimaging

Antibodies

Antibodies used	Only standard secondary antibodies needed here including HRP goat anti-mouse IgG (Fisher Cat-No 31430); rabbit anti-Myc antibody (Rockland); donkey anti-rabbit (Invitrogen); mouse anti-His (ThermoFisher Scientific)

Validation	Secondary antibodies used herein. Anti-NANP serum generated as part of the study was validated for anti-CSP specificity as described in lines 132-158

Animals and other research organisms

Policy information about studies involving animals; ARRIVE guidelines recommended for reporting animal research, and Sex and Gender in Research

Laboratory animals	BALB/cj and C57BL/6 mice from Jackson Laboratories (male and female; both 4-6 weeks old) are specified.
Wild animals	N/A
Reporting on sex	The sex of the animals is described for each relevant experiment.
Field-collected samples	N/A
Ethics oversight	Animal studies were approved by the University of Washington Institutional Animal Care and Use Committee under protocol 4317-01.

Note that full information on the approval of the study protocol must also be provided in the manuscript.
